# Supplementary material for: Phospho-Specific Flow Cytometry Reveals Signaling Heterogeneity in T-Cell Acute Lymphoblastic Leukemia Cell Lines
Source: Cells. 2022 Jun 29;11(13):2072. doi: 10.3390/cells11132072 (PMC9266179; doi:10.3390/cells11132072)
Supplement: Supplementary file 1 [file cells-11-02072-s001.zip › cells-1774237-SI.pdf]

Phospho-specific flow cytometry reveals signaling heterogeneity in T-cell acute lymphoblastic leukemia cell lines

Perbellini O et al.
Supplementary Material

Supplementary Tables

Supplementary Table S1. Antibodies used for the detection of protein expression

Antibody	Clone	Fluorochrome	Manufacturer
CD1a	HI149	FITC	BD Biosciences
CD2	S5.2	V450	BD Biosciences
cCD3	SK7	PE-Cy7	BD Biosciences
mCD3	UCHT1	APC	BD Biosciences
CD5	L17F12	PerCp-Cy5.5	BD Biosciences
CD7	M-T701	APC	BD Biosciences
CD8	SK1	FITC	BD Biosciences
CD45	HI30	PerCp-Cy5.5	BD Biosciences
CD127	HIL-7R-M21	PE-Cy7	BD Biosciences
CD184	12G5	PE	BD Biosciences

cCD3: cytoplasmic CD3; mCD3: membrane CD3.

Supplementary Table S2. Modulators used in the study

Modulator	Concentration	Manufacturer
Unmodulated	-	-
H2O2	3,3 mM	Sigma-Aldrich
PMA	400 nM	Sigma-Aldrich
IL-7	10 ng/mL	Peprotech
CXCL12	100 ng/mL	Peprotech

Phospho-specific flow cytometry reveals signaling heterogeneity in T-cell acute lymphoblastic leukemia cell lines

Perbellini O et al.
Supplementary Material

Supplementary Table S3. Antibodies used for the detection of phosphoproteins

Antibody	Clone	Fluorochrome	Manufacturer
pSTAT5 (Y694)	47/Stat5(pY694)	Alexa Fluor 488	BD Biosciences
pp38 (T180/Y182)	36/p38(pT180/pY182)	Alexa Fluor 488	BD Biosciences
pRb (S780)	J146-35	Alexa Fluor 488	BD Biosciences
pLck (Y505)	MOL171	Alexa Fluor 488	BD Biosciences
pNF-κB p65 (S529)	K10-895.12.50	PE	BD Biosciences
pErk1/2 (T202/Y204)	20A	PE	BD Biosciences
pZAP70 (Y292)	J34-602	PE	BD Biosciences
pZAP70 (Y319)	17A/P-ZAP70	PE	BD Biosciences
pAkt (S473)	M89-61	Alexa Fluor 647	Cell Signaling Technologies
pJNK (Thr183/Tyr185)	G9	Alexa Fluor 647	Cell Signaling Technologies
pSTAT3 (S727)	49/p-Stat3	Alexa Fluor 647	BD Biosciences
CD45 (for HDs)	HI30	PerCp-Cy5.5	BD Biosciences
cCD3*	UCHT1	PE-Cy7	BD Biosciences

*: cytoplasmic CD3

Supplementary Table S4. Staining panels for the detection of phosphoproteins

Alexa Fluor 488	PE	PerCp-Cy5.5	Pe-Cy7	Alexa Fluor 647	Near-IR
pSTAT5	pNF-kB p65	CD45	cCD3	pAkt	LIVE/DEAD
pp38	pErk1/2	CD45	cCD3	pJNK	LIVE/DEAD
pRb	pZAP70 (Y292)	CD45	cCD3	pSTAT3	LIVE/DEAD
pLck	pZAP70 (Y319)	CD45	cCD3	-	LIVE/DEAD

cCD3: cytoplasmic CD3.

Phospho-specific flow cytometry reveals signaling heterogeneity in T-cell acute lymphoblastic leukemia cell lines

Perbellini O et al.
Supplementary Material

Supplementary Figures

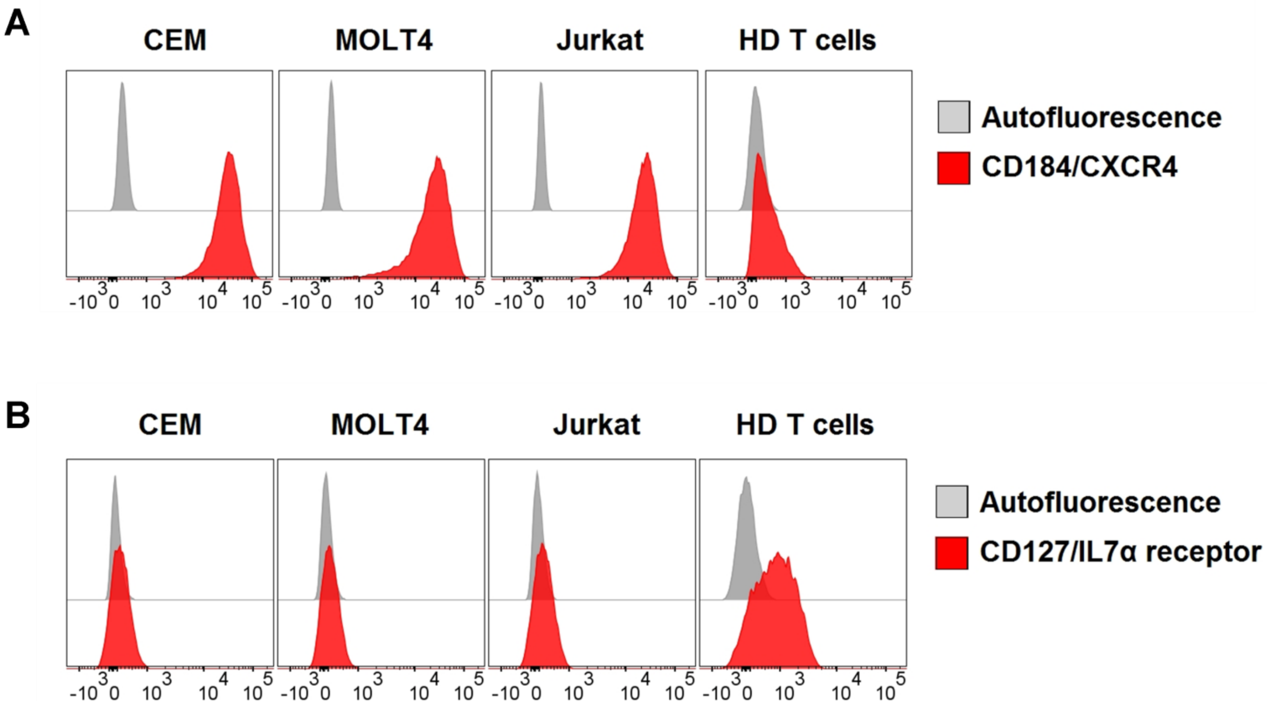

Supplementary Figure S1. Comparison of expression of receptors for physiological stimuli. Surface expression of CD184/CXCR4 (**A**) and CD127/IL7α (**B**) on T-ALL cell lines and T cells from healthy donor (HD) was evaluated by flow cytometry, using autofluorescence as control. Data are representative of two independent experiments.

Phospho-specific flow cytometry reveals signaling heterogeneity in T-cell acute lymphoblastic leukemia cell lines

Perbellini O et al.
Supplementary Material

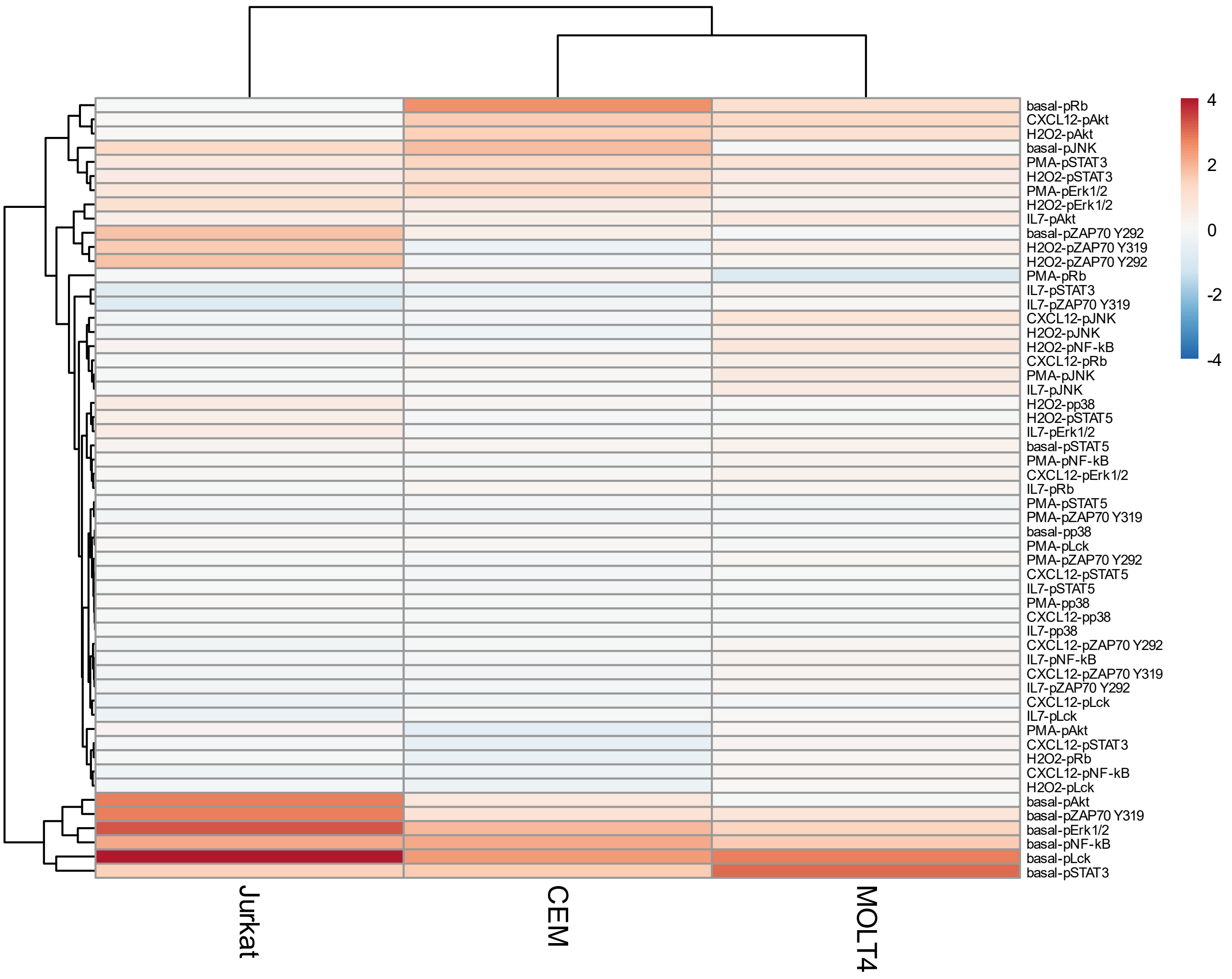

Supplementary Figure S2. Unsupervised hierarchical clustering analysis of all the analyzed nodes. Unsupervised hierarchical clustering analysis of all the analyzed nodes (n=55) within the T-ALL cell lines CEM, MOLT4, and Jurkat.

Phospho-specific flow cytometry reveals signaling heterogeneity in T-cell acute lymphoblastic leukemia cell lines

Perbellini O et al.
Supplementary Material

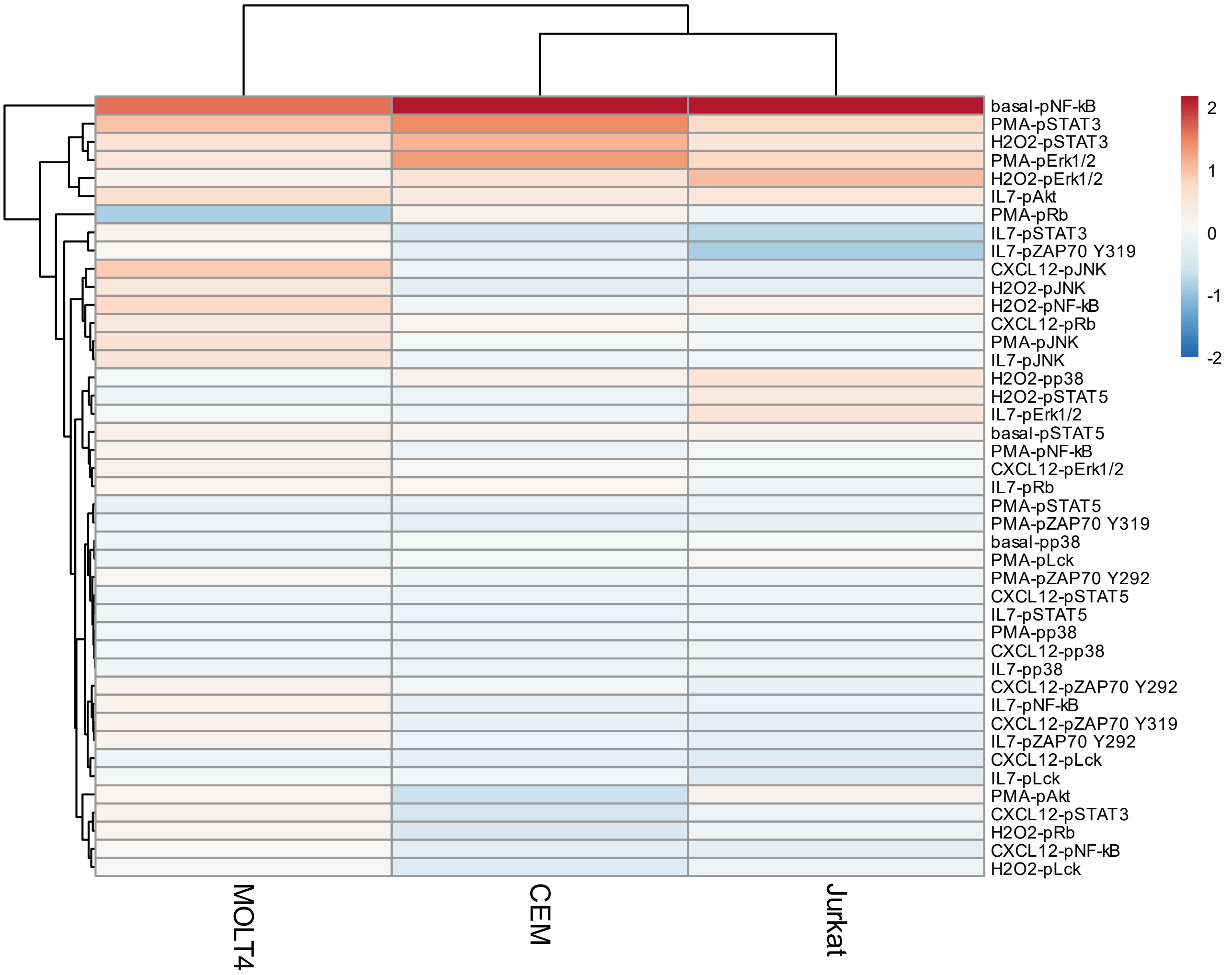

Supplementary Figure S3. Unsupervised hierarchical clustering analysis on selected nodes. Unsupervised hierarchical clustering analysis of nodes (n = 43) following exclusion of nodes showing higher variation (σ^2 of $\log_2$ values >0.5) across samples within the T-ALL cell lines CEM, MOLT4, and Jurkat.
